# Characterization of genomic instability-related genes predicts survival and therapeutic response in lung adenocarcinoma

**DOI:** 10.1186/s12885-023-11580-0

**Published:** 2023-11-16

**Authors:** Shuyang Li, Wei Wang, Huihan Yu, Siyu Zhang, Wenxu Bi, Suling Sun, Bo Hong, Zhiyou Fang, Xueran Chen

**Affiliations:** 1 School of Basic Medicine, Anhui Medical University, No. 81, Meishan Road, Hefei, 230032 Anhui China; 2 Hefei Cancer Hospital of CAS, Institute of Health and Medical Technology, Hefei Institutes of Physical Science, Chinese Academy of Sciences (CAS), No. 350, Shushan Hu Road, Hefei, 230031 Anhui China

**Keywords:** Lung adenocarcinoma, Tumor immune microenvironment, Genetic signature associated with genomic instability, Chemotherapy, Immune characteristic

## Abstract

**Background:**

Lung adenocarcinoma (LUAD) is the most common subtype of non-small cell lung cancer (NSCLC) and is the leading cause of cancer death worldwide. Its progression is characterized by genomic instability. In turn, the level of genomic instability affects the prognosis and immune status of patients with LUAD. However, the impact of molecular features associated with genomic instability on the tumor microenvironment (TME) has not been well characterized. In addition, the effect of the genes related to genomic instability in LUAD on individualized treatment of LUAD is unknown.

**Methods:**

The RNA-Sequencing, somatic mutation, and clinical data of LUAD patients were downloaded from publicly available databases. A genetic signature associated with genomic instability (GSAGI) was constructed by univariate Cox regression, Lasso regression, and multivariate Cox regression analysis. Bioinformatics analysis investigated the differences in prognosis, immune characteristics, and the most appropriate treatment strategy among different subtypes of LUAD patients. CCK-8 and colony formation verified the various effects of Etoposide on different subtypes of LUAD cell lines. Cell-to-cell communication analysis was performed using the “CellChat” R package. The expression of the risk factors in the GSAGI was verified using real-time quantitative PCR (qRT-PCR) and Immunohistochemistry (IHC).

**Results:**

We constructed and validated the GSAGI, consisting of five genes: ANLN, RHOV, KRT6A, SIGLEC6, and KLRG2. The GSAGI was an independent prognostic factor for LUAD patients. Patients in the high-risk group distinguished by the GSAGI are more suitable for chemotherapy. More immune cells are infiltrating the tumor microenvironment of patients in the low-risk group, especially B cells. Low-risk group patients are more suitable for receiving immunotherapy. The single-cell level analysis confirmed the influence of the GSAGI on TME and revealed the Mode of action between tumor cells and other types of cells. qRT-PCR and IHC showed increased ANLN, RHOV, and KRT6A expression in the LUAD cells and tumor tissues.

**Conclusion:**

This study confirms that genes related to genomic instability can affect the prognosis and immune status of LUAD patients. The GSAGI we identified has the potential to guide clinicians in predicting clinical outcomes, assessing immunological status, and even developing personalized treatment plans for LUAD patients.

**Supplementary Information:**

The online version contains supplementary material available at 10.1186/s12885-023-11580-0.

## Introduction

Lung cancer is the leading cause of cancer-related deaths worldwide, with non-small cell lung cancer (NSCLC) accounting for about 85% of cases and lung adenocarcinoma (LUAD) being the most common subtype [1]. Despite advancements in clinical treatments and individualized therapies, the 5-year overall survival (OS) rate for LUAD remains low at around 16% [2]. Therefore, searching for appropriate therapeutic strategies for LUAD patients is a research hotspot.

Genomic instability, which increases the likelihood of acquiring mutations [3], is a hallmark of most cancers, including lung cancer [4], and is caused by factors such as smoking, air pollution, and radiation exposure. The total number of somatic mutations can be quantified by tumor mutation burden (TMB) [5], associated with poor prognosis in certain cancers, including NSCLC [6]. Abnormalities in transcriptional and post-transcriptional regulation are related to genomic instability, suggesting the potential of molecular markers as a quantitative measure of genomic instability [7]. For example, Habermann et al. analyzed the gene expression profiles of 48 breast cancer specimens and identified 12 genes characterized by genomic instability [8]. Geng et al. established a gene tag of seven genes associated with genomic instability that could predict the prognosis of patients with LUAD [9]. The genomic instability is crucial for the occurrence and development of lung adenocarcinoma. It is necessary to explore further the effects of genes derived from genomic instability on the progression of LUAD.

Tumor microenvironment (TME) refers to the internal and external environment during tumors’ occurrence, growth, and metastasis. TME comprises immune cells, stromal cells, and various cytokines [10], among which immune cells play a crucial role in tumor development. For instance, current studies suggest that tumor-infiltrating B lymphocytes (TIL-B) can promote anti-tumor immunity through their unique antigen-presenting mode, leading to the persistence of an immune “hot” TME involving T cells, bone marrow cells, and natural killer cells [11]. In addition, the expression of immune checkpoints on tumor cells helps them evade host immune surveillance [12], whereas inhibiting immune checkpoints with immune checkpoint inhibitors (ICIs) can restore immune cell function. The expression levels of PD1 and PD-L1 significantly affect LUAD patients’ response to ICI therapy [13]. Moreover, the roles of specific chemokines and their corresponding receptors in the immune therapy response for LUAD are complex. They may affect tumor immune cell infiltration, immune regulation, tumor growth, and metastasis [14]. However, the association between genes derived from genomic instability and the composition of TME, expression of immune checkpoints and chemokines, and ICI efficacy remains ambiguous.

In this study, we constructed a genetic signature associated with genomic instability (GSAGI), including five factors, ANLN, RHOV, KRT6A, SIGLEC6, and KLRG2. The GSAGI showed favorable prognostic results for LUAD patients. The Nomogram model created based on this was more accurate. In addition, patients in the two subgroups distinguished with GSAGI may have differences in TME due to different intercellular communication patterns. And significant differences in the composition of chemokine and immune checkpoint expression profiles may also influence the optimal treatment of LUAD patients.

## Materials and methods

### Data collection and preprocessing

The RNA-Sequencing, somatic mutation, and clinical data of LUAD patients were downloaded from the TCGA (https://portal.gdc.cancer.gov) database. The Ensemble (http://www.ensembl.org/) database was used to annotate mRNA. 499 clinical samples with mRNA expression profile data and survival data were randomly divided into a “training set” (n = 251) and a “testing set” (n = 248) by the R package “caret.“ In addition, LUAD patients with paired RNA-seq data and clinical data in GSE31210 (n = 246), GSE30219 (n = 85), GSE50081 (n = 127), GSE42127 (n = 133), and GSE41271 (n = 182) from GEO (https://www.ncbi.nlm.nih.gov/geo/) database are independent external testing sets. The detailed information on these patients is listed in Table S1 (Additional file 1: Table S1). GSE126045 (n = 16) as an independent external testing set for immunotherapy prediction. The R package “DESeq2” was used to screen for differential genes.

### Functional enrichment analysis

KEGG is a knowledge base for systematic analysis of gene functions, linking genomic information with higher order functional information [15]. It is now one of the most utilized biological databases because of its practical values. Together with an improved annotation procedure for KEGG Orthology assignment, an increasing number of eukaryotic genomes have been included in KEGG for better representation of organisms in the taxonomic tree [16, 17]. The R package “clusterProfiler” and “org.Hs.eg.db” perform the GO and KEGG (www.kegg.jp/kegg/kegg1.html) pathway enrichment analysis of all candidate differential genes [18]. The threshold for significant pathway enrichment was set to a P-value < 0.05 and visualized using R software’s “ggplot2” package.

### Construction and validation of the GSAGI

First, univariate Cox regression analysis was performed on the candidate differential genes in the TCGA-LUAD training set using the R package “survival”, to screen for genes that were significantly correlated (P-value < 0.05) with survival in LUAD patients. The genes with prognostic value were then filtered by the least absolute shrinkage and selection operator (LASSO) algorithm, and the penalty parameters were adjusted by 10-fold cross-validation with the R packages “glmnet” and “survivor”. Finally, the multivariate Cox regression analysis on the genes screened by the LASSO algorithm to obtain the best candidate genes. We constructed the following risk score formula using the expression levels (expr) of the best candidate differential genes and the regression coefficients (coef) from the multivariate Cox regression analysis:$Risk score={\sum }_{i=1}^{n}expri*coefi$

The expri represents the expression level of the ith gene, and coefi represents the coefficient of the ith gene. Patients were divided into high-risk and low-risk groups using the median risk score of the samples in each data set as the threshold value. Survival curves were plotted by the Kaplan-Merier method. The R packages “survival” and “survminer” were used to compare the survival of patients in the high-risk and low-risk groups. P-value < 0.05 indicates significance. The predictability of the prognostic model was assessed by plotting time-dependent receptor operating characteristic (ROC) curves with R package “survROC”.

### Nomogram model construction and validation

In the TCGA-LUAD set, we performed a multivariate COX regression analysis on patients’ age, gender, disease stage, smoking history, EGFR mutation status, and grouping information based on the GSAGI. P-value < 0.05 indicates statistical significance. Finally, the GSAGI and the disease staging were used to construct a Nomogram model as a quantitative analysis tool. The R package “rms” [19] was used to produce it. Calibration curves and ROC curves validated the predictive performance of this Nomogram model.

### Genomic enrichment analysis (GSEA)

To explore this gene signature’s impact on LUAD patients’ biological function, we downloaded “c5.all.v7.0.entrez.gmt” from the MSigDB database (http://www.gsea-msigdb.org/gsea/downloads.jsp) for GSEA annotation. The R package “enrichplot” selected pathways [20].

### Predicting chemotherapy response levels

The R package “pRRophetic” [21] inferred the sensitivity of chemotherapeutic agents in LUAD patients. The RNA expression profiles of 68 LUAD cell lines were obtained from the Broad Institute’s Cancer Cell Line Encyclopedia (CCLE, https://portals.broadinstitute.org/ccle/) [22]. IC50 values of LUAD cell lines to chemotherapeutic drugs were obtained from Genomics of Drug Sensitivity in Cancer (GDSC, https://www.cancerrxgene.org/) [23].

### Cell viability assay

LUAD cells were seeded in 96-well plates at 5,000 cells per well and incubated overnight. The cells were treated with different concentrations of Etoposide (20, 40, 60, 80, 100, 120, 150 and 180µM) followed by 24-hour incubation. Next, the cells were treated with WST-8 from CCK-8 (NCM Biotech, Suzhou, China) for 0.5-1 h; then, their viability was measured by detecting the absorbance at OD 450 nm.

### Colony formation assay

In a 6-well plate, 3000 LUAD cells were plated in triplicate and incubated overnight, then grown for ten days in a growth medium with Etoposide (0, 0.5, 1.0, 1.5, and 2.0µM). We then washed the cells thrice with PBS, fixed them in cold methanol for 20 min, and cleaned and stored them. Settled cell colonies were visualized by incubating the cells with 0.5% (w/v) crystal violet for 0.5 h. Extra crystal violet was removed by washing with PBS. Visible colonies formed by LUAD cell growth were identified by ImageJ version 1.8.0.112 software. Colony numbers would reflect cell survival and proliferation.

### Evaluation of immune cell infiltration

The ESTIMATE algorithm calculated the immune score and tumor purity [24]. The R package “CIBERSORT” evaluated the infiltration of 22 kinds of immune cells in LUAD patients with different risk scores [25]. The differences in the degree of B-cell infiltration between patients in high-risk and low-risk groups were compared using the TIMER database (http://timer.cistrome.org/) [26].

### Prediction of the response to immunotherapy

The Immunophenoscore (IPS) was obtained according to The Cancer Immunome Atlas (TCIA, https://tcia.at/home), and the higher the IPS, the more responsive the patients were to immunotherapy [27]. In addition, the TIDE website (http://tide.Dfci.harvard.edu/) predicts the degree of response to immunotherapy in patients with different risk scores based on transcriptomic data from patients [28].

### Single-cell data quality control and identification of major cell types

Twenty-six LUAD samples from the GSE148071 dataset were used for single-cell level analysis. We ended up with 4593 cells for downstream analysis after removing cells with gene expression values below 200 or above 5000 and discarding cells with mitochondrial content higher than 10%. “NormalizeData” and “ScaleData” functions in the “Seurat” R package normalized the expression matrix. Then the “FindVariable” function was applied to select the first 2000 variable genes and perform principal component analysis. The first ten main components and a resolution of 0.5 were used for cell clustering by the “FindClusters” function. The “FindVariable” function generated groups of differentially expressed genes (DEGs). We manually annotated cell types for each cell cluster based on the normalized expression of DEGs in conjunction with typical markers from the CellMarker (http://xteam.xbio.top/CellMarker/) website [29] (Additional file 2: Table S2). And visualization was performed by uniform manifold approximation and projection (UMAP).

### Cell-cell communication analysis

The R package “CellChat” determines cellular communication between tumor cells and other cell types [30]. The “netVisual bubble” function allows us to observe the differences in receptors and ligands of tumor cells interacting with other cells between low and high-grouped patients.

### Cell lines and RNA extraction and real-time quantitative PCR (qRT-PCR)

The human lung adenocarcinoma cell lines (A549, PC-9, NCI-H1299, and NCI-H1975) and the human bronchial epithelial cell line BEAS-2B were gifted by Zhiyou Fang’s group at the Center for Basic Medicine, Institute of Health and Medical Technology, Hefei Institute of Material Science, Chinese Academy of Sciences. In this study, all cell lines were cultured in RPMI-1640 containing 1% (100×) streptomycin/penicillin and 10% FBS. The culture environment was humid, with a temperature of 37 °C and 5% CO2. We used an RNA preparation kit (TransGen Biotech, 220 Beijing) to extract total RNA from the cell lines. The reverse transcription reaction system consisted of total RNA, 2 µg; Anchored Oligo(dT)18, 1 µL; 2*TS Reactiob Mix, 1 µL; TransScript RT/RI Enzyme Mix, 10 µL; and RNase-free Water, added to the total system for a total of 20 µL. The cDNA was prepared by placing the reverse transcription system in HiScript II Q RT SuperMix for qPCR (+ gDNA wiper) (Vazyme Biotech, Nanjing, NJ), after 42 °C, 5 min; 85 °C, 5 s. ChamQ Universal SYBR qPCR Master Mix (Vazyme Biotech, Nanjing) was used for quantitative RT-PCR analysis. RT-PCR analysis was performed in 3 replicates using a X 960 Real-time PCR (Heal Force). The three-step amplification procedure we used was as follows: The first step, denaturation at 94 ℃ for 30 s; The second step (40 cycles): denaturation at 95 ℃ for 10 s, annealing at 55–60 ℃ for 20 s, and extension at 72 ℃ for 20 s. The third step: terminate the extension at 72 ℃ for 20 s. Finally, the melting curve was output. Relative mRNA expression was calculated using 2^−ΔΔCT^ software. β-actin was used as an internal control gene. Primers used in the study (Additional file 3: Table S3) were purchased from Sangong Bioengineering Co Ltd (Shanghai, China).

### Tissue samples and immunohistochemistry (IHC)

Tissue sections from six LUAD patients were collected at the Hefei Cancer Hospital of the Chinese Academy of Sciences, and performed IHC staining on normal and tumor samples. Tissue sections were deparaffinized and rehydrated through graded ethanol. Citrate buffer was used for antigen repair. 3% H2O2 was used to block endogenous peroxidase activity. 5% bovine serum albumin (BSA) was used to block non-specific binding. The sections were then incubated with primary antibodies against the protein of interest overnight at 4 °C. The primary antibody was detected with a secondary antibody conjugated with horseradish peroxidase (HRP) and visualized using diaminobenzidine (DAB) as a substrate. The sections were counterstained with hematoxylin and mounted with coverslips. The primary antibodies we used were: mouse monoclonal anti-human ANLN antibody (1:200; Santa Cruz, sc-271,814) mouse monoclonal anti-human RHOV antibody (1:200; Santa Cruz, sc-515,072), and rabbit monoclonal anti-human KRT6A antibody (1:200; Proteintech, 10590-1-AP). To quantify the positive staining of immunohistochemical (IHC) slides, we used ImageJ software [31].

### Statistical analysis and visualization

R version 4.1.2, GraphPad Prism 9.0, GraphPad Prism version 9.0, and ImageJ version 1.8.0.112 software were used for statistical analysis and visualization. |Log2foldchange (FC)| ≥ 1 and adjusted P-value < 0.05 as the threshold for differentially expressed genes. P-value < 0.05 was considered statistically significant. Kaplan-Meier curves were used to determine differences in survival between patient groups, and Log-rank tests were used to calculate statistical significance. Non-normally distributed continuous variables were analyzed by the Wilcox test method. Pearson’s correlation coefficient is used to explore the correlation between two continuous variables. A one-way ANOVA analysis was utilized to evaluate the differences between more than two groups.

## Results

### The GSAGI accurately predicts prognosis in LUAD patients

**Fig. 1:**
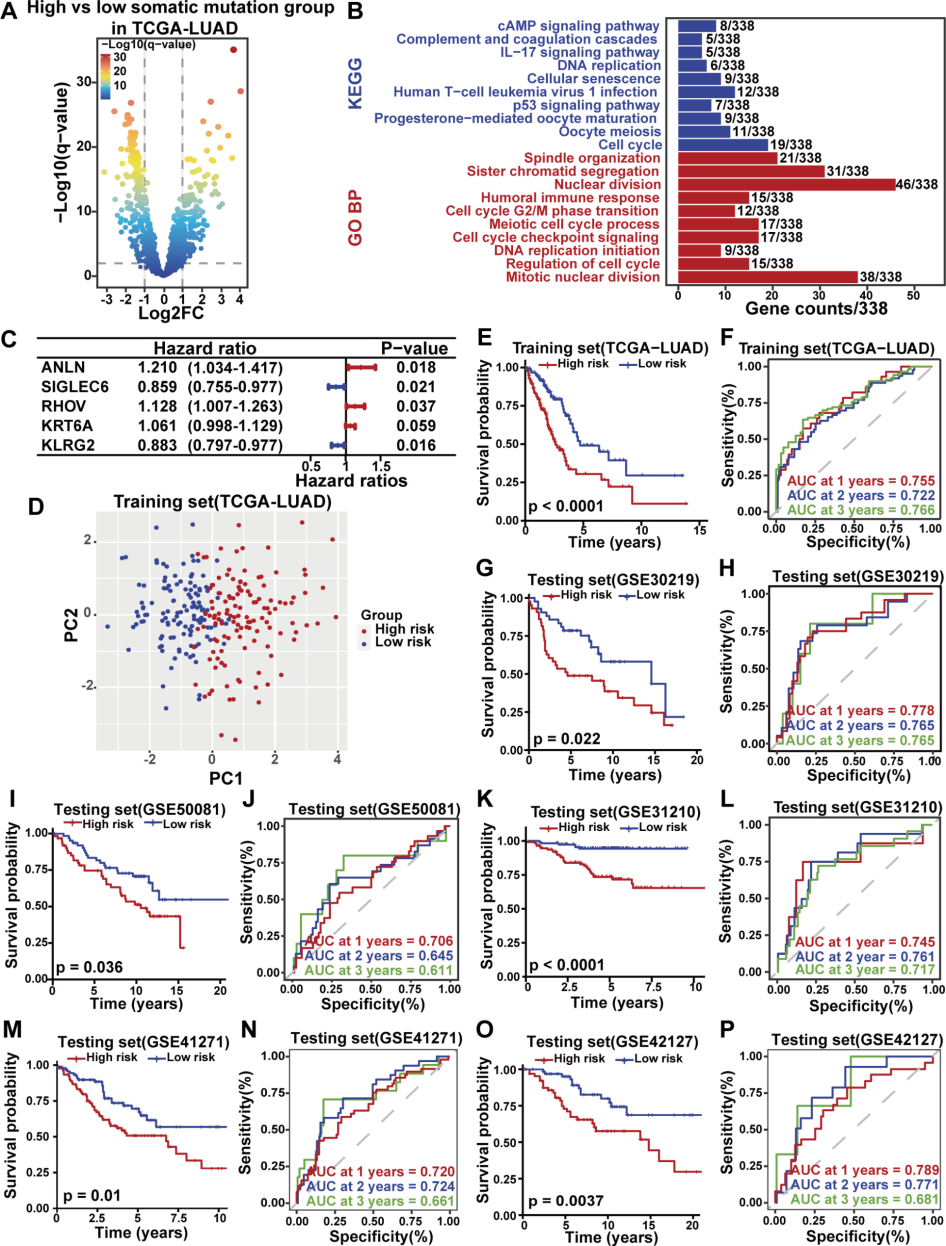
The GSAGI predicts prognosis in LUAD patients. **A** Volcano map of differentially expressed genes in LUAD patients in high and low somatic mutation groups. **B** Pathway enrichment analysis of GO and KEGG for 347 candidate differential genes, p-values of pathways shown are all < 0.05. **C** Hazard ratio and P-value of constituents involved in multivariate Cox regression. **D** PCA of TCGA-LUAD training set to distinguish between high-risk and low-risk groups. **E, G, I, K, M, O** Kaplan-Meier survival curves in the TCGA-LUAD training set **(E)**, GSE30219 testing set **(G)**, GSE50081 testing set **(I)**, GSE31210 testing set **(K)**, GSE41271 testing set **(M)**, and GSE42127 testing set **(O)** for patients in the high-risk and low-risk groups differentiated by the GSAGI. **F, H, J, L, N, P** ROC curves for patients in the TCGA-LUAD training set **(F)**, GSE30219 testing set **(H)**, GSE50081 testing set **(J)**, GSE31210 testing set **(L)**, GSE41271 testing set **(N)**, and GSE42127 testing set **(P)**. AUCs at 1, 2, and 3 years are shown in the figures

Firstly, the cumulative number of somatic mutations per LUAD patient in the TCGA database was calculated to screen for genes associated with genomic instability. Samples with TMB ≥ 10 (n = 98) were considered the high-level somatic mutation group. Samples with TMB < 1.43 (n = 98) were considered the low-level somatic mutation group to ensure consistency in sample size. We next selected genes that co-significantly differentially expressed between LUAD samples and normal tissue samples as genes associated with LUAD (|Log2FC| ≥ 1 and adjusted P-value < 0.05) in the GSE31210 dataset of the GEO platform and TCGA-LUAD set (Additional file 4: Figure S1A). Of the 1261 genes associated with LUAD, 177 genes were significantly up-regulated in the high-level somatic mutation group, while 170 genes were significantly down-regulated (|Log2FC| ≥ 1 and adjusted P-value < 0.05, Fig. 1A). GO and KEGG pathway enrichment analysis found the functions of these 347 candidate differential genes were significantly associated with genomic instability (Fig. 1B), such as Cell cycle, G2/M phase transition, Meiotic cell cycle process, P53 signaling pathway, and DNA replication, et al. We further investigated the prognostic impact of these genes on LUAD patients. 131 genes of predictive value were identified by univariate COX regression analysis on patients in the TCGA-LUAD training set (Additional file 5: Table S4). Using the LASSO algorithm, nine genes were validated and selected (Additional file 4: Figure S1B, S1C), and further identified five genes comprising the GSAGI (Fig. 1C) by multivariate COX regression analysis. Multivariate Cox regression analysis retained the factor KRT6A, which has added value for predicting the prognosis of LUAD patients. The risk score for each LUAD patient can be obtained from this GSAGI as Risk score = (0.190805622) * ANLN expression + (0.120404221) * RHOV expression + (0.059586055) * KRT6A expression + (-0.152189144) * SIGLEC6 expression + (-0.124976472) * KLRG2 expression.

In the training and testing sets of TCGA, patients in the high-risk group had a shorter survival time and a higher proportion of dead samples. The heat maps showed that ANLN, RHOV, and KRT6A expression levels increased with increasing risk scores, while SIGLEC6 and KLRG2 decreased (Additional file 4: Figure S1D, S1E). In addition, principal component analysis (PCA) showed that LUAD patients with different risk stratification could be divided into two subgroups explicitly (Fig. 1D and Additional file 4: Figure S1F). Kaplan-Meier curve plotted for the TCGA-LUAD training set showed that patients in the low-risk group had more prolonged OS than those in the high-risk group (Fig. 1E). The AUCs of the ROC curves at 1, 2, and 3 years were 0.755, 0.722, and 0.766 (Fig. 1F). In the TCGA-LUAD testing set (n = 248) and TCGA-LUAD set (n = 499), low-risk patients also showed a significantly better prognosis (Additional file 4: Figure S1G, S1I), and ROC curves show good predictive performance (Additional file 4: Figure S1H, S1J). Finally, we performed an independent external validation using five datasets from the GEO database, including GSE31210, GSE30219, GSE50081, GSE42127, and GSE41271. LUAD patients were similarly divided into high and low-risk groups using the median risk score as the threshold. Kaplan-Meier curves for each set demonstrated consistent trends with previous results (Fig. 1G and I K, 1 M, 1O). The AUCs of the ROC curves at 1, 2, and 3 years for each external validation set are more significant than 0.6 (Fig. 1H J, 1 L, 1 N, 1P). These results suggest that our constructed GSAGI has high accuracy and sensitivity in predicting the prognosis of LUAD patients.

### Exploration of mutational patterns in different risk levels LUAD patients

**Fig. 2:**
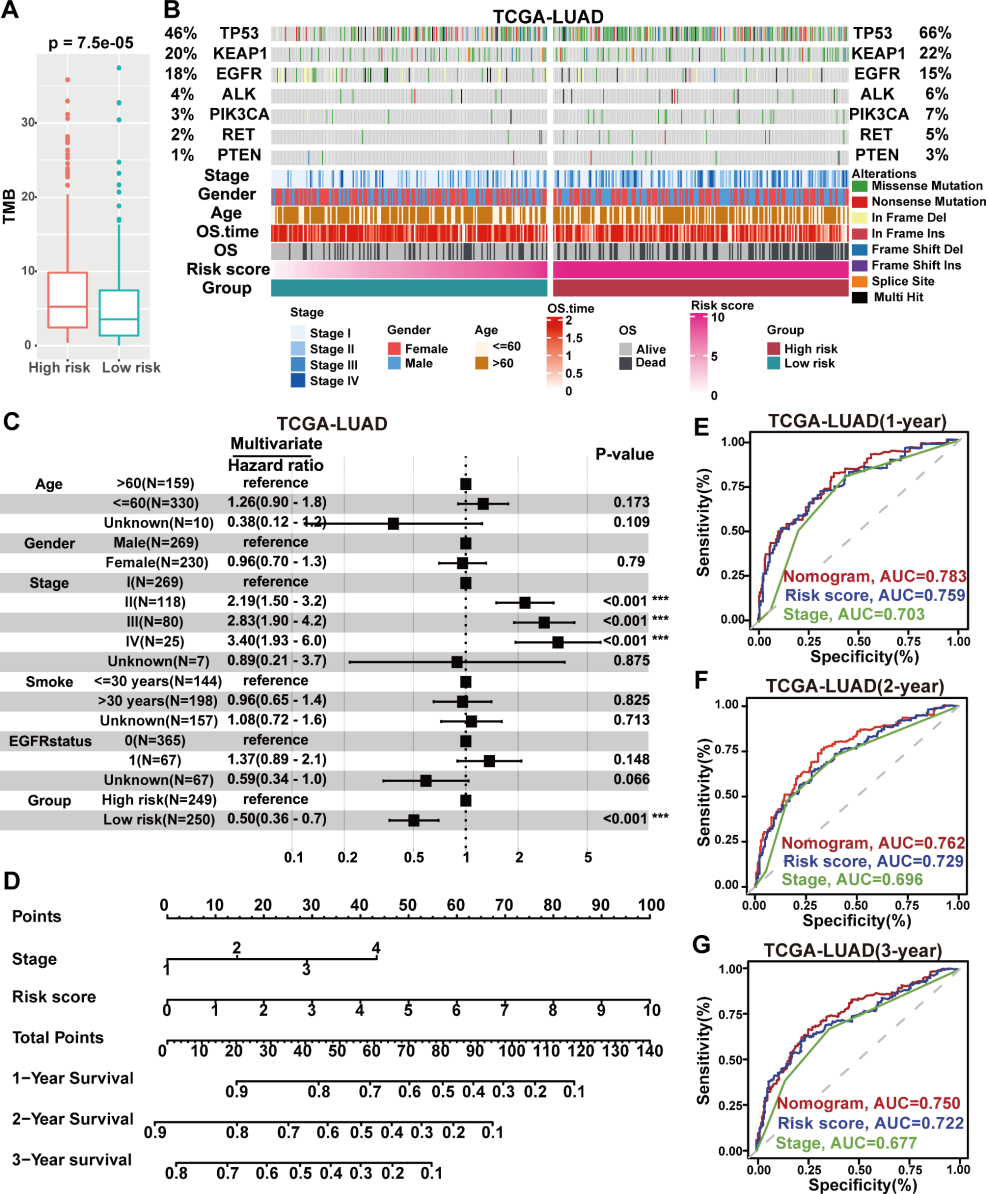
Explore the mutation status of LUAD patients and construct and validate a Nomogram. **A** Overall somatic mutation levels among TCGA-LUAD patients in different risk strata. **B** The waterfall diagram demonstrates the mutation status of patients with different clinical characteristics and risk stratifications in the TCGA-LUAD set. **C** Forest plots of age, gender, stage, smoking history, EGFR mutation status, and risk stratification for multivariate COX regression analysis. ***P < 0.001. **D** Nomogram model for predicting OS in LUAD patients. **E-G** ROC curves show the predictive efficiency of the Nomogram model, risk score values, and stage for 1-year **(E)**, 2-year **(F)**, and 3-year **(G)** OS in LUAD patients

We compared overall somatic mutation levels among TCGA-LUAD patients in different risk strata. Patients in the high-risk group exhibited significantly higher genomic instability (Fig. 2A). Next, seven important mutation patterns in LUAD were highlighted for exploration. TP53 mutations were more frequent in high-risk group patients (Fig. 2B). As one of the most common genetic variations in LUAD, TP53 mutations are significantly associated with higher mutation levels and poorer prognosis in patients. TP53 mutations can suppress tumor immunogenicity, reducing patient response to immunotherapy, such as ICIs [32]. The slightly higher mutations in KEAP1, ALK, PIK3CA, RET, and PTEN in the high-risk group of patients also suggest that patients in the high-risk group may have a worse prognosis.

We further conducted a multivariate Cox regression analysis of the clinical features (age, gender, staging, smoking history, EGFR mutation status) of patients in the TCGA-LUAD set and different risk groups distinguished by risk scores (Fig. 2C). The results showed that only staging and grouping were independent factors affecting the prognosis of LUAD patients. Therefore, the nomogram model is based on patients’ staging and risk scores in the TCGA-LUAD set (Fig. 2D). The ROC analysis confirmed the effectiveness of the Nomogram model, which demonstrated higher predictive accuracy survival in LUAD patients compared to risk scores or stage alone (Fig. 2E F, and 2G). The calibration curve also confirmed the reliability of the Nomogram model (Additional file 4: Figure S2A). In conclusion, the significant differences in the number of mutations and mutation patterns between patients in the high- and low-risk groups may be one of the reasons for the better survival of patients in the low-risk group. The nomogram model constructed based on GSAGI can predict the survival of LUAD more accurately.

### LUAD patients in the high-risk group distinguished by GSAGI are better suited for chemotherapy

**Fig. 3:**
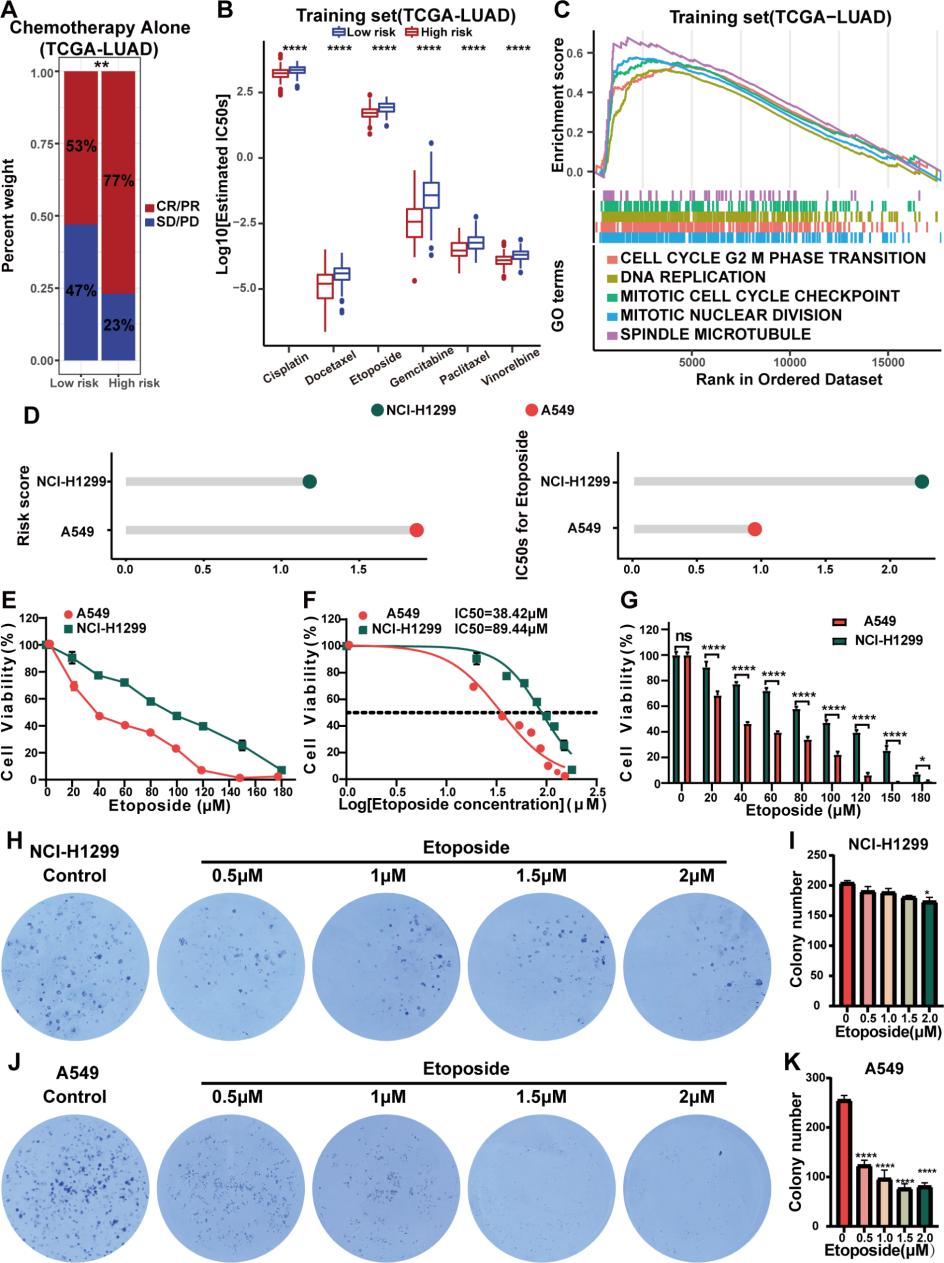
Prediction of chemotherapy response level in LUAD patients and cell lines validation. **A** Histogram of the percentage of patients in the high and low-risk groups who received chemotherapy only in the TCGA-LUAD set who responded to treatment. **P < 0.01. **B** Boxplots reflect the differences in the degree of response to chemotherapy drugs between patients in the high-risk and low-risk groups in the TCGA-LUAD training set. ****P < 0.0001. **C** GSEA analysis shows signaling pathways significantly enriched in patients in the high-risk groups in the TCGA-LUAD training set. **D** Risk scores and IC50s to Etoposide in A549 and NCI-H1299 cell lines. **E-F** An CCK-8 assay was used to evaluate the viability of A549 and NCI-H1299 cells under different concentrations of Etoposide (20, 40, 60, 80, 100, 120, 150, and 180 µM) for 24 h. GraphPad Prism was used to analyze and visualize the data from the LUAD cells viability assay and the IC50 of Etoposide. **G** The LUAD cells viability of NCI-H1299 vs. A549. *P < 0.05 and ****P < 0.0001. **H, J** Colony formation assay of NCI-H1299 **(H)** and A549 **(J)** cell lines was performed to detect the colony formation ability. **I, K** Quantitative analysis of colony formation formed by LUAD cells was performed using ImageJ, followed by GraphPad Prism visualization. *P < 0.05, and ****P < 0.0001 vs. control group

We subsequently investigated the relationship between the GSAGI and chemotherapy response rates in LUAD patients. The TCGA-LUAD dataset included 103 LUAD patients who received chemotherapy alone and had matched treatment response information. They were categorized into high-risk and low-risk groups using the median risk scores as a threshold. However, the GSAGI failed to accurately distinguish the prognosis of these two patient groups (Additional file 4: Figure S3A). Interestingly, the high-risk group exhibited significantly higher response rates to chemotherapy drugs (Fig. 3A). This result suggests that LUAD patients in the high-risk group may be more suitable for chemotherapy. Still, age, gender, disease stage, and genetic mutations may influence OS and limit better outcomes.

Next, we predicted the response of LUAD patients to commonly used chemotherapy drugs in clinical practice [33]. In the TCGA-LUAD training, TCGA-LUAD testing, and TCGA-LUAD sets, the high-risk group showed a trend of higher sensitivity to standard chemotherapy drugs (Cisplatin, Docetaxel, Etoposide, Gemcitabine, Paclitaxel, and Vinorelbine) (Fig. 3B and Additional file 4: Figure S3B, S3C). The results of GSEA showed that the high-risk group was significantly enriched in signaling pathways such as the cell cycle, DNA replication, and spindle microtubules (Fig. 3C and Additional file 4: Figure S3D, S3E). It is reported that the most commonly used chemotherapy drugs for LUAD (Paclitaxel, Docetaxel, and Vinorelbine) exert anti-tumor effects by binding to microtubules. Abnormal activation of microtubules may lead to the proliferation and invasion of tumor cells, thereby increasing the sensitivity of tumor cells to microtubule-targeted drugs [34]. Moreover, Risk scores were significantly positively correlated with the expression of a commonly used anticancer drug target: DNA topoisomerase IIA (TOP2A), and significantly negatively correlated with the expression of multidrug resistance protein 1 (ABCB1) [35] (Additional file 4: Figure S3F, S3G) may explain why high-risk group patients are more suitable for chemotherapy.

Meanwhile, we investigated the expression of five characteristic genes in 68 LUAD cell lines from the CCLE database. We identified three low-risk group cell lines (NCI-H2291, NCI-H1755, and NCI-H1573) and three high-risk group cell lines (NCI-H1355, NCI-H2009, and NCI-H2030) (Additional file 4: Figure S3H and Additional file 6: Table S5). The GDSC database showed that all three high-risk group cell lines were significantly more sensitive to Etoposide (Additional file 4: Figure S3I and Additional file 6: Table S5), verified a positive correlation between GSAGI score and chemo-sensitive.

In addition, based on results from the CCLE and GDSC sites, the A549 cell line had a higher risk score and higher drug sensitivity to Etoposide (Additional file 6: Table S5 and Fig. 3D), which we experimentally verified. The CCK-8 assay showed a dose-dependent decrease in cell viability at 24 h when 20, 40, 60, 80, 100, 120, 150, and 180 µM of Etoposide were treated with both A549 and NCI-H1299 (Fig. 3E F). And the cell viability of NCI-H1299 was significantly higher than that of A549 after treatment with the same dose of Etoposide (Fig. 3G). Next, we added different concentrations of Etoposide into LUAD cell lines. The results showed that the number of clones formed by the A549 cell line was more significantly inhibited than NCI-H1299 (Fig. 3H and I J, and 3 K). That is, the same dose of Etoposide more significantly inhibited the survival and proliferation of A549 cells. These results demonstrate that LUAD patients with higher scores by GSAGI may be better suited to receive chemotherapy treatment.

### The GSAGI affects the immune characteristics of LUAD

**Fig. 4:**
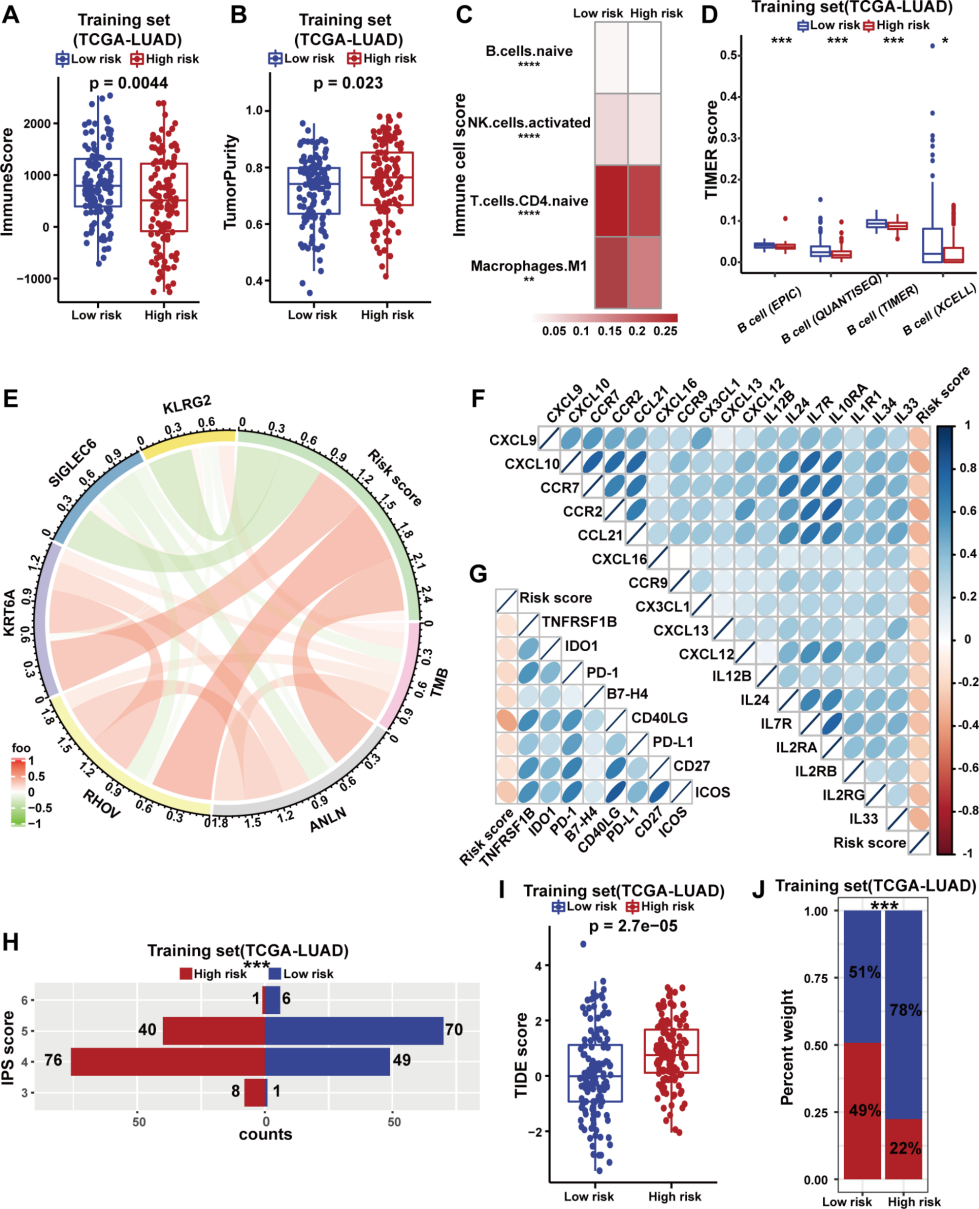
The GSAGI predicts the immune characteristics of LUAD. **A** ESTIMATE algorithm evaluates the immune score of patients in the high-risk and low-risk groups in the TCGA-LUAD training set. **B** ESTIMATE algorithm evaluates the tumor purity of patients in the TCGA-LUAD training set. **C** The CIBERSORT algorithm assesses the differences in the degree of infiltration of B cells naive, NK cells activated, T cells CD4 naive, and Macrophages M1 between the high-risk and low-risk groups in the TCGA-LUAD set. **P < 0.01 and ****P < 0.0001. **D** Four algorithms in the TIMER database assess the differences in the degree of infiltration of B cells between the high-risk and low-risk groups in the TCGA-LUAD training set. *P < 0.05 and ***P < 0.001. **E** Positive correlations between TMB levels and the expression of ANLN, RHOV, KRT6A, and KLRG2, and some negative correlations with SIGLEC6 expression in LUAD patients. **F-G** Correlation between risk scores of LUAD patients and the expression of 17 chemokines and their receptors **(F)** and the expression of 7 immune checkpoint molecules **(G)**. The colors represent Pearson correlation coefficients, and the sizes of the ellipses represent the P-values. **H** Two-way bar graphs show IPS for patients in the high-risk and low-risk groups in the TCGA-LUAD training set. ***P < 0.001. **I** Comparison of TIDE scores between patients in the high-risk and low-risk groups in the TCGA-LUAD training set. **J** The percentage bar graph compares the different response statuses of patients receiving immunotherapy in the high-risk and low-risk groups in the TCGA-LUAD training set. ***P < 0.001. Red indicates that the patient responded to ICI treatment, blue indicates non-response

Using the ESTIMATE algorithm, the patients in the low-risk group exhibited higher immune scores (Fig. 4A and Additional file 4: Figure S4A, S4B), indicating that low-risk group patients may have higher immune cell infiltration. Meanwhile, patients in the high-risk group showed higher tumor purity (Fig. 4B and Additional file 4: Figure S4C, S4D). Next, the CIBERSORT algorithm was used to describe the infiltration of different immune cells in patients with different risk groups. Low-risk group patients showed higher levels of infiltration of immature B cells, activated dendritic cells, immature CD4 T cells, and M1 macrophages (Fig. 4C). TIL-B can promote anti-tumor immunity through their unique antigen presentation and play a critical role in maintaining a “hot” tumor microenvironment (including T cells, bone marrow cells, and natural killer cells). We further used four methods in the TIMER website to evaluate the level of B cell infiltration in different risk-stratified LUAD patients. The result confirmed that B cell infiltration levels in the low-risk group were significantly higher (Fig. 4D and Additional file 4: Figure S4E, S4F).

Surprisingly, there was some positive correlation between the risk scores obtained from the GSAGI and the risk scores of LUAD patients (Fig. 4E). LUAD patients with low-risk scores had lower levels of TMB and higher levels of immune cell infiltration. Patients in the low-risk group may have a “hot” tumor immune microenvironment. At the same time, we found some positive correlations between TMB levels and the expression of ANLN, RHOV, KRT6A, and KLRG2, and some negative correlations with SIGLEC6 expression in LUAD patients (Fig. 4E). This somewhat suggests that the five GSAGI genes we screened may influence TMB levels in LUAD patients.

Chemokines and their receptors are involved in regulating immune cell localization and function to promote the proliferation and survival of immune cells. Correlation analysis showed a significant negative correlation between the expression of some chemokines and their receptors and risk score (Fig. 4F) [36]. More importantly, there was a significant negative correlation between the presentation of joint immune checkpoints (ICOS, CD27, PD-L1, CD40LG, B7-H4, PD-1, IDO1, TNFRSF1B) and risk score (Fig. 4G) [37]. Next, the IPS was used to explore the differences in immunogenicity among LUAD patients in different risk groups. Low-risk group LUAD patients showed significantly higher immunogenicity (Fig. 4H and Additional file 4: Figure S4G, S4H). Furthermore, we used the TIDE website to predict that the low-risk group had significantly lower TIDE scores (Fig. 4I and Additional file 4: Figure S4I, S4J). At the same time, the proportion of low-risk group patients who could respond to ICI treatment was significantly higher (Fig. 4J and Additional file 4: Figure S4K, S4L). Finally, we used the dataset GSE126045 from the GEO database of LUAD patients who received immunotherapy for validation. We divided 16 LUAD patients into low- and high-risk groups using the median of GSAGI score. Among the eight high-risk group LUAD patients, only one responded to immunotherapy, whereas half of the patients in the low-risk group responded to immunotherapy (Additional file 4: Figure S4M). The lack of significant differences may be due to the small sample size of the testing set. The above results suggest that GSAGI can robustly assess the immune characteristics of LUAD patients and the degree of responsiveness to immunotherapy.

#### Intercellular communication affects the Tumor immune microenvironment in LUAD

**Fig. 5:**
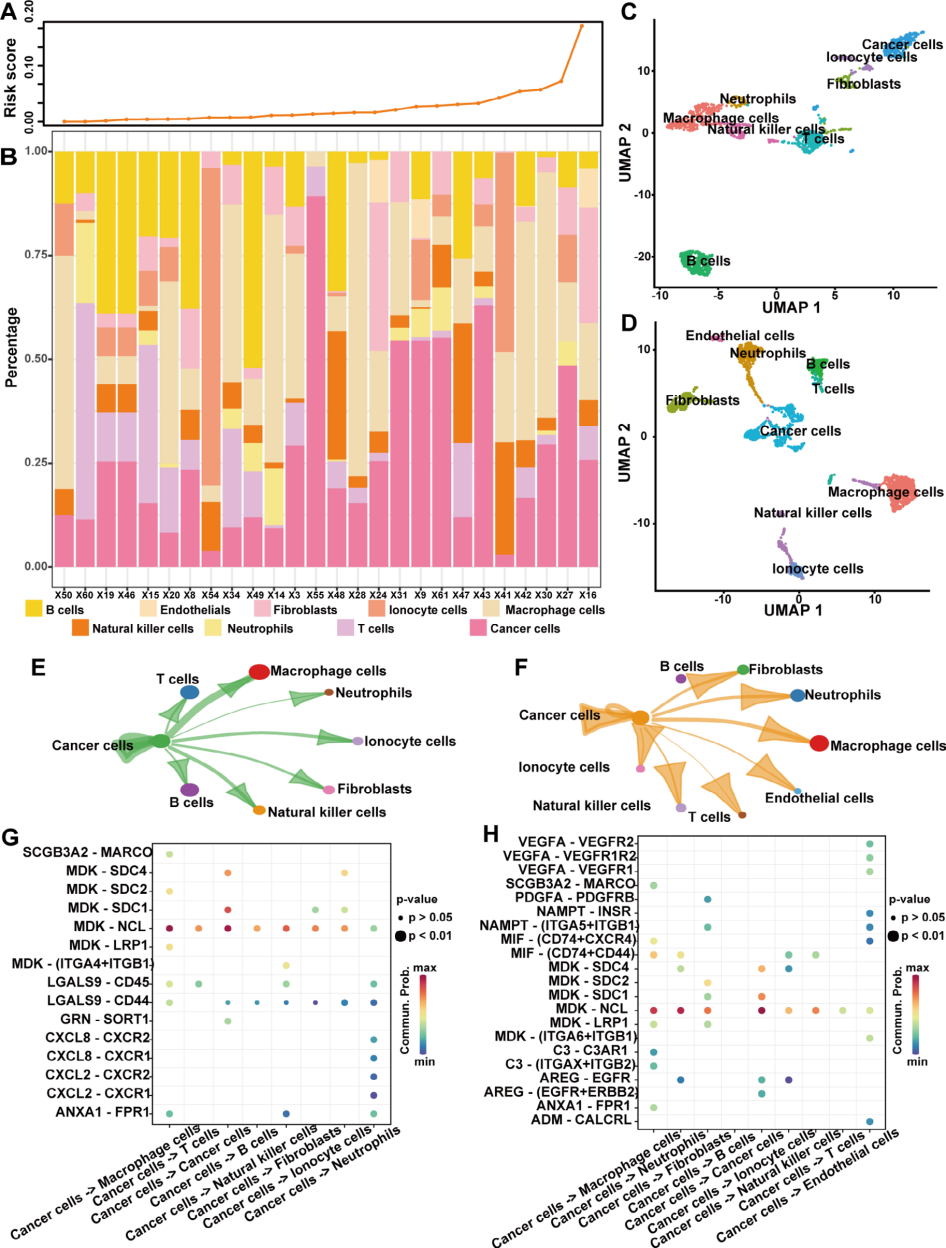
Intercellular communication affects the tumor immune microenvironment in LUAD. **A** Risk scores of 26 LUAD patients. **B** Histograms showing the proportion of cells in the TME of different patients. **C-D** UMAP plots show the single-cell mapping of low (C) and high (D) subgroups. **E-F** Interactions between tumor cells and other cells in the TME for low **(E)** and high **(F)** subgroups. **G-H** Activation pathways of tumor cells interacting with other cells in the TME of low **(G)** and high **(H)** subgroups

Single-cell sequencing technology can characterize intra-tumor heterogeneity better than conventional tissue sequencing. We further explored the relationship between GSAGI and tumor immune microenvironment at single-cell resolution. In the GSE148071 dataset, we normalized the single-cell sequencing data of 26 LUAD patients and calculated a risk score for each cell by the GSAGI. Further, we assessed the risk score of each LUAD patient (the average of the risk scores of all cells in each sample) and classified the patients into low and high groups (Fig. 5A). UMAP visualized 11 cell clusters in the low and 14 in the high groups. (Additional file 4: Figure S5A, S5B). We assigned one of the nine major cell types to each cluster by standardized expression of typical markers on the “CellMarker” website. (Additional file 4: Figure S5C, S5D). We pooled groups of cells of the same type together for analysis (Fig. 5C and D). Interestingly, an increased risk score may result in more tumor cells, fewer B cells, and fewer T cells in the TME (Fig. 5B). And the difference in the proportions of tumor, B, and T cells was statistically significant in the samples with high and low subgroups (Additional file 4: Figure S5E). That is, an increase in GSAGI scores is associated with a decrease in immune cells.

In addition, we examined cellular interactions in the TME of patients with two subtypes of lung adenocarcinoma. A higher number of cells in high-group patients may lead to more robust intercellular communication compared to low-group patients (Additional file 4: Figure S5F, S5G). However, we further focused on tumor cells and could see that tumor cells interacted more strongly with B cells, T cells, NK cells, and macrophages in the low group (Fig. 5E F). Understanding the interactions between tumor cells and immune cells helps to understand the mechanisms of cancer progression and metastasis, so we further explored the receptor-ligand interaction Mode of action between tumor cells and other types of cells. We identified immunosuppressive pathways: the MDK-NCL can inhibit B-cell receptor signaling [38], and ANXA1-FPR1 recruits and polarizes bone marrow-derived macrophages [39]. Moreover, the mode of action of LGALS9-CD45 between tumor cells and T cells and the mode of action of LGALS9-CD44 between tumor cells and B cells in low subgroups of patients belong to the immunosuppressive pathway, which can be used as a potential immunotherapeutic target in the low group [40]. More importantly, the CXCL-CXCR pathway between tumor cells and neutrophils in the low group was enriched in immunoreactive isoforms (Fig. 5G H), which may also serve as a target for immunotherapy [41]. The above results further revealed the role of the GSAGI in the tumor immune microenvironment.

### Practical application of risk factors in the GSAGI to LUAD

**Fig. 6:**
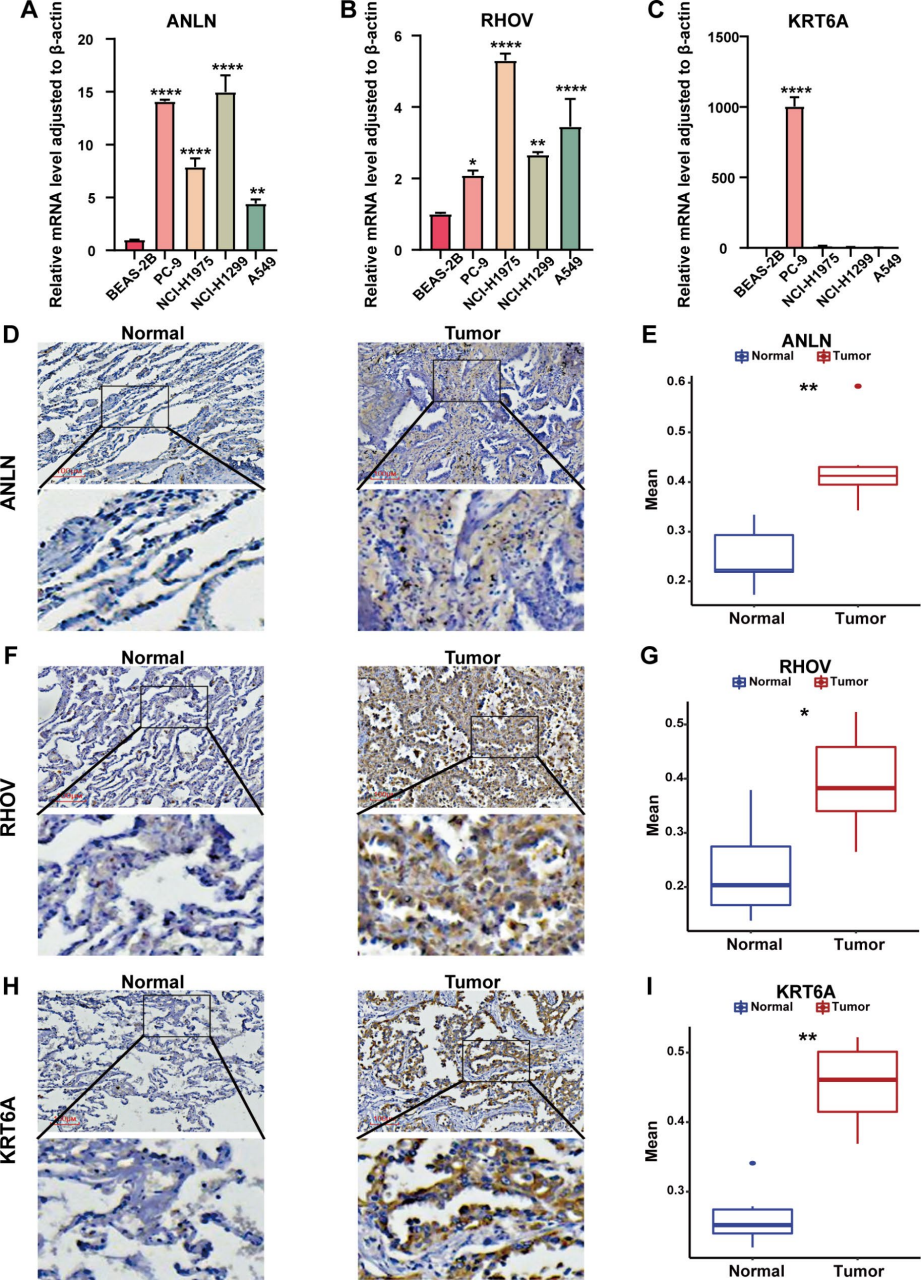
Characterized gene expression validation in cell lines and LUAD tissues. **A-C** Histograms of different transcript levels of ANLN **(A)**, RHOV **(B)**, and KRT6A **(C)** in LUAD cell lines and human normal lung epithelial cells BEAS-2B. **D, F, H** IHC of ANLN **(D)**, RHOV **(F)**, and KRT6A **(H)** in tissues of LUAD patients. **E, G, I** Statistics of IHC positivity in tissues of 6 LUAD patients. *P < 0.05 and **P < 0.01

The results of real-time PCR (RT-PCR) showed that the mRNA levels of ANLN and RHOV were significantly higher in four LUAD cell lines (A549, PC-9, NCI-H1975, and NCI-H1299) than in the human lung epithelial cell line BEAS-2B (Fig. 6A and B). However, KRT6A only significantly upregulated in the PC-9 cell line (Fig. 6C). To explore the expression of three risk factors in this gene feature in LUAD patient tissues, tumor tissues, and normal tissues from six LUAD patients at the Hefei Cancer Hospital of the Chinese Academy of Sciences were collected for IHC validation. ImageJ software calculated the positivity rate, and the results confirmed that ANLN, RHOV, and KRT6A were all significantly upregulated in the tumor samples (Fig. 6D and I).

## Discussion

The development of LUAD is highly complex and closely related to the abnormal expression of specific genes. In the past few decades, many therapeutic targets and predictive biomarkers have been identified due to the continuous development of high-throughput sequencing technologies [42]. LUAD is a type of tumor with high genomic instability, and genomic instability as the basis of cancer characteristics can accelerate the acquisition of genetic diversity and promote the formation of various cancer characteristics. Genomic instability is crucial for the progression and recurrence of cancer and is associated with poor prognosis, metastasis, and treatment resistance [43]. Therefore, an in-depth understanding of the molecular mechanisms that affect the genomic instability of LUAD can provide more accurate biomarkers for diagnosing and treating tumors. However, there are no reliable biomarkers to detect the association between genes related to genomic instability and the tumor microenvironment and immune features of LUAD patients.

In this study, we used the FDA (Food and Drug Administration)’s TMB > = 10 criteria for high TMB. The 98 LUAD samples with TMB > = 10 from the TCGA database were used as the high TMB group, and the 98 samples corresponding to the lowest TMB values were used as the low TMB group. We identified 347 genes that are associated with the occurrence of lung adenocarcinoma (LUAD) and genomic instability. Based on this, we established a GSAGI consisting of five genes that can more accurately predict the prognosis of LUAD patients. We found that patients in the high-risk group had higher levels of genomic instability. This is consistent with the conclusion of Owada-Ozaki et al. that NSCLC patients with lower TMB levels may have a better prognosis [8]. Further, we found that patients in the high-risk group were more suitable for chemotherapy, and the abnormal activation of microtubules, microfilaments, and other pathways in patients may cause better chemotherapy results. Moreover, the predicted results from the TIDE website suggest that LUAD patients in the low-risk group are better suited to receive immunotherapy. This contradicts the previous view that immunotherapy efficacy is better in LUAD patients with higher TMB levels [44]. Surprisingly, a study by Nie W et al. in 2020 showed that NSCLC patients with low TMB may because of significantly higher levels of Th1 and Th17 cells more suitable anti-PD-1/PD-L1 immunotherapy [45].

Further studies found that LUAD patients in the low-risk group had higher levels of immune cell infiltration, especially B-cell infiltration. The significantly higher levels of immune cell infiltration in low-risk group may have contributed to the persistence of a “hot” tumor immune microenvironment. Thus low-risk group showed to be more suitable for ICI treatment. In addition, PD-L1 expression and TMB were not significantly correlated in most cancer subtypes, and the non-overlapping effects of PD-L1 expression and TMB on the response rate to PD-1/PD-L1 inhibitors could be widely used to classify immunosubtypes of cancers. PD-L1 expression and TMB may each provide information for the use of ICI [46]. Previous studies have also shown that PD-L1 is a crucial indicator for ICI treatment in LUAD patients [47]. The negative correlation between the risk score and the expression of immune checkpoints, chemokines, and their receptors may also be one of the reasons why LUAD patients in the low-risk group are more suitable for ICI treatment. Accordingly, we believe that although high TMB levels may increase the chance of the immune system recognizing and attacking tumor cells, it is not the only factor affecting patients’ immune response. Immune checkpoints, chemokines and their receptors, as well as TME are also important factors influencing the efficacy of ICI in patients with LUAD. At the same time, the GSAGI was shown to possibly better identify LUAD patients who are more suitable to receive immunotherapy.

Findings at the single-cell level suggest that this GSAGI may influence the TME of LUAD. Tumor cells tended to receive higher GSAGI scores than immune cells. CellChat analyses showed stronger interactions between tumor and B cells, T cells, and macrophage cells in low-grouped patients. The receptor-ligand mode of action between tumor cells and immune cells in LUAD patients (MDK-NCL, ANXA1-FPR1, LGALS9-CD45, LGALS9-CD44, and CXCL-CXCR et al.) may serve as targets for immunotherapy.

The high expression of ANLN, RHOV, and KRT6A is associated with significantly worse survival in LUAD patients (Additional file 4: Figure S6A, S6B, and S6C). ANLN is a myosin-binding protein whose expression level and localization are regulated by the cell cycle, and it is an essential component of cytokinesis [48]. The loss of ANLN may affect the progression of the cell cycle. ANLN has been reported to be significantly upregulated in various tumors [49]. And genomic instability shown due to alterations in the cell cycle is one of the characteristics of many cancers [50]. This may explain the existence of some degree of positive correlation between ANLN and TMB. RHOV is an atypical member of the Ras superfamily of small GTPases. It regulates the cell cycle, promotes cell differentiation, and affects cell adhesion and migration [51]. It has been reported that RHOV activates the JNK/c-Jun pathway, leading to the metastasis of LUAD. Similarly, The effect of RHOV on the cell cycle may partially influence genomic instability in LUAD patients, resulting in a weak positive correlation between RHOV expression and TMB. KRT6A, a member of the keratin family, has been shown to influence the epithelial-mesenchymal transition [52], and its overexpression promotes the proliferation and invasion of NSCLC cells. Epithelial cells acquire a mesenchymal phenotype during epithelial-mesenchymal transition, and recent studies have linked epithelial-mesenchymal transition to many cellular functions including genomic instability, cancer cell drug resistance, and metabolic adaptations [53]. Moreover, a study by Chantapet et al. found that fragment 19 of another member of the keratin family, KRT19 (CYFRA 21 − 1), can serve as a serum biomarker for diagnosing NSCLC [54]. Therefore, it is necessary to explore further whether KRT6A can be used as a serum biomarker for diagnosing NSCLC. According to our research, SIGLEC6 is a protective factor for LUAD (Additional file 4: Figure S6D). Current research indicates that SIGLEC6 is expressed explicitly in B cells, monocytes, and placental trophoblasts [55]. SIGLEC6 belongs to the sialic acid-binding immunoglobulin-like lectin family, a family of immune regulatory receptors [56]. The interaction between sialylated glycans and SIGLEC6 can modulate immune cell function during tumorigenesis, resulting in an immunosuppressive tumor microenvironment. Another gene in the GSAGI, KLRG2, also showed a favorable impact on the prognosis of LUAD patients (Additional file 4: Figure S6E). KLRG2 plays a vital role in carbohydrate recognition and binding [57]. KLRG2 contains a C-type lectin/C-type lectin-like domain (CTL/CTLD), and its receptor is expressed on various immune cells. In addition, this domain is involved in cell adhesion, migration, pathogen recognition, and intercellular signaling. We evaluated the expression of KLRG2 using the Tumor Immune Single-cell Hub 2 (TISCH2) database, which focuses on the tumor microenvironment. In an NSCLC dataset (GSE99254) on the GEO platform, KLRG2 was mainly expressed on Mono/Macro cells (Additional file 4: Figure S6F). The results of CIBERSORT analysis showed significantly higher levels of M2 macrophages, resting mast cells, and monocyte infiltration in the high KLRG2 expression group of LUAD patients (Additional file 4: Figure S6G). These findings suggest that KLRG2 may significantly affect regulating the tumor immune microenvironment. However, the two current protective factors in GSAGI, SIGLEC6, and KLRG2, have not been studied for genomic instability. The negative correlation between SIGLEC6 and TMB levels and a certain degree of positive correlation between KLRG2 and TMB levels found in our study necessitates further confirmation. The association between SIGLEC6 and KLRG2 and genomic instability necessitates in-depth exploration.

In summary, the GSAGI identified in this study provides a direction for prognosis prediction and individualized treatment of LUAD patients. Although these studies have revealed significant findings, there are also some limitations. Firstly, our analysis based on public databases may need to be more convincing. Although the GSAGI performed well in several external testing sets, prospective studies in the future are necessary to validate the feasibility of this gene signature. Secondly, although IHC validation confirmed the high expression of ANLN, RHOV, and KRT6A in LUAD patients, we need a large clinical sample to increase the reliability of the results. The specific mechanisms by which these risk factors promote cancer also need further exploration. Finally, the prediction of personalized treatment for LUAD patients with different risk stratification needs to be further confirmed. The in-depth exploration of molecular features associated with genomic instability will provide direction for diagnosing and personalized treating LUAD.

## Conclusion

This study identified the GSAGI of five genes (ANLN, RHOV, KRT6A, SIGLEC6, and KLRG2) by screening genes associated with genomic instability. It was able to accurately and sensitively predict the prognosis of LUAD patients. The GSAGI can predict the tumor immune microenvironment in LUAD, which may be because tumor cells and immune cells interact in different ways between different subtypes of LUAD patients. In conclusion, our study offers the possibility of predicting the survival of LUAD patients and provides a basis for individualized treatment plans for LUAD patients.

### Electronic supplementary material

Below is the link to the electronic supplementary material.

Supplementary Material 1

Supplementary Material 2

Supplementary Material 3

Supplementary Material 4

Supplementary Material 5

Supplementary Material 6

## Data Availability

The data supporting this study’s findings are openly available, which can be retrieved. from the TCGA-LUAD dataset from TCGA (https://portal.gdc.cancer.gov), GSE GSE31210, GSE30219, GSE50081, GSE42127, GSE41271,GSE126045, and GSE148071 datasets from GEO (https://www.ncbi.nlm.nih.gov/geo/) .

## Abbreviations

TCGA: The cancer genome atlas
GEO: Gene expression omnibus data base
LASSO: Least absolute shrinkage and selection operator
RNA-seq: RNA sequencing
OS: Overall survival
FC: Fold change
FDR: False discovery rate
ROC: Receiver operating characteristic
GO: Gene ontology
KEGG: Kyoto encyclopedia of genes and genomes
TIDE: Tumor immune dysfunction and exclusion
GSEA: Gene set enrichment analysis
MSigDB: Molecular Signatures Database
DMEM: Dulbecco’s modified Eagle’s medium
qRT-PCR: Real-time quantitative PCR
OD: Optical density
IHC: Immunohistochemistry
PCA: Principal component analysis
CR: Complete response
PR: Partial response
SD: Stable disease
PD: Progressive disease
HR: Hazard ratio
TMB: Tumor mutation burden
TME: Tumor microenvironment
LUAD: Lung adenocarcinoma
GSAGI: Genetic signature associated with genomic instability
NSCLC: Non-small cell lung cancer
ICI: Immune checkpoint inhibitor
TIL-B: Tumor-infiltrating B lymphocytes
FDA: Food and Drug Administration
UMAP: Uniform manifold approximation and projection
