# Supplementary material for: Characterization of genomic instability-related genes predicts survival and therapeutic response in lung adenocarcinoma
Source: BMC Cancer. 2023 Nov 16;23:1115. doi: 10.1186/s12885-023-11580-0 (PMC10655275; doi:10.1186/s12885-023-11580-0)
Supplement: Supplementary file 3 — Supplementary Material 3 [file 12885_2023_11580_MOESM3_ESM.docx]

**Additional file 3: Table S3. Primer design sequence for RT-PCR.**

| Gene | Forward primer (5’-3’ on plus strand) | Reverse primer (5’-3’ on plus strand) |
| --- | --- | --- |
| β-actin | CATCCGCAAAGACCTGTACG | CCTGCTTGCTGATCCACATC |
| ANLN | TCCTCAAGTGCTGATGATGC | TTCTCCCTTGGATGGAACTG |
| RHOV | CAGCCTCATCGTCAGCTACA | CGAAGTCGGTCAAAATCCTC |
| KRT6A | CCAAGGCAGACACTCTCACA | TCATATTGGGCCTTGACCTC |
