# Supplementary material for: Characterization of genomic instability-related genes predicts survival and therapeutic response in lung adenocarcinoma
Source: BMC Cancer. 2023 Nov 16;23:1115. doi: 10.1186/s12885-023-11580-0 (PMC10655275; doi:10.1186/s12885-023-11580-0)
Supplement: Supplementary file 4 — Supplementary Material 4 [file 12885_2023_11580_MOESM4_ESM.docx]

Additional file 4

**Title**

Characterization of genomic instability-related genes predicts survival and therapeutic response in lung adenocarcinoma.

**Author Information**

Shuyang Li1,2,#, Wei Wang1,2,#, Huihan Yu1,2, Siyu Zhang2, Wenxu Bi2, Sunling Sun2, Bo Hong2, Zhiyou Fang1,2,*, Xueran Chen1,2,*

1School of Basic Medicine, Anhui Medical University, No. 81, Meishan Road, Hefei, Anhui, 230032, China

2Hefei Cancer Hospital of CAS; Institute of Health and Medical Technology, Hefei Institutes of Physical Science, Chinese Academy of Sciences (CAS), No. 350, Shushan Hu Road, Hefei, Anhui, 230031, China

# These authors contributed equally to this work.

*Corresponding author: Prof. Zhiyou Fang ([z.fang@cmpt.ac.cn](mailto:zyfang@cmpt.ac.cn)), and Dr. Xueran Chen ([xueranchen@cmpt.ac.cn](mailto:xueranchen@cmpt.ac.cn)).

**This file includes:**

Figures. S1-S6


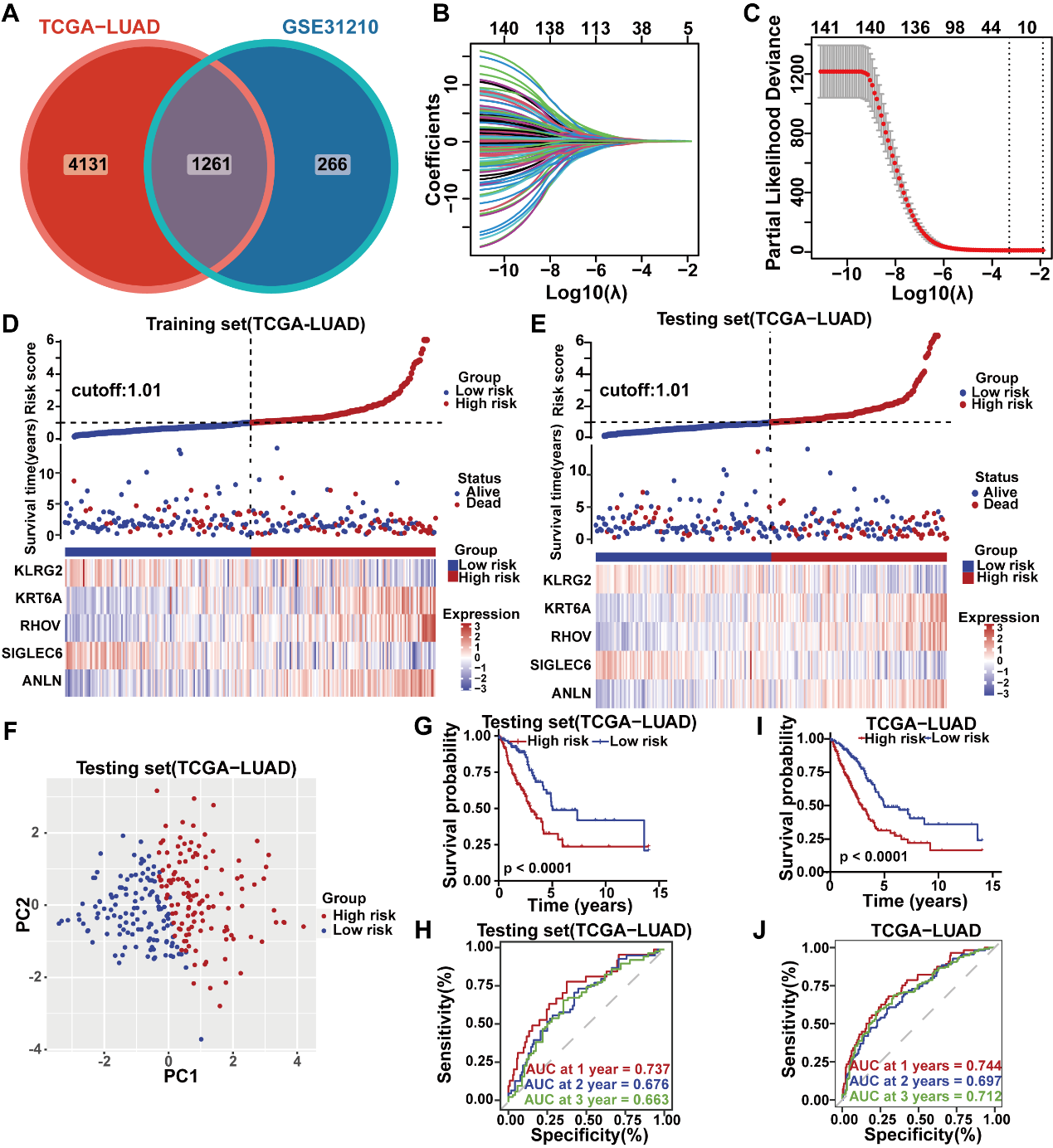


**Additional file 4: Figure S1.** **Risk scores obtained from the GSAGI predict survival in LUAD patients.**

(**A**) Venn diagram showed 1261 genes associated with LUAD in both the TCGA-LUAD and GSE31210 datasets. (**B**) Ten-time cross-validation for tuning parameter selection in the LASSO model. (**C**) LASSO coefficient profiles of 131 mRNAs. (**D**) Scatter plot of risk scores versus survival status and heat map of gene expression in the TCGA-LUAD training set. (**E**) Scatter plot of risk scores versus survival status and heat map of gene expression in the TCGA-LUAD testing set. (**F**) PCA of TCGA-LUAD testing set to distinguish between high-risk and low-risk groups. (**G, I**) Kaplan-Meier survival curves in the TCGA-LUAD testing set (G) and TCGA-LUAD set (I) for patients in the high-risk and low-risk groups differentiated by the GSAGI. (**H, J**) ROC curves for patients in the TCGA-LUAD testing set (H) and TCGA-LUAD set (J). AUCs at 1, 2, and 3 years are shown in the figures.


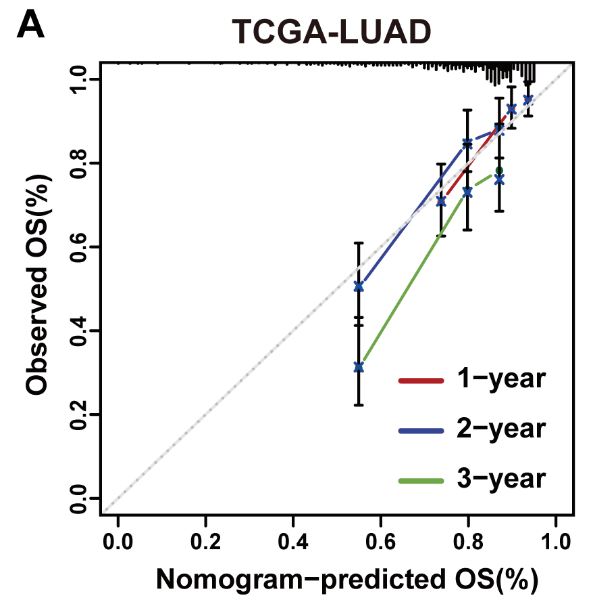


**Additional file 4: Figure S2.** **Validation of the predictive efficiency of the Nomogram model.**

(**A**) Calibration curves showing the accuracy of the Nomogram model for predicting OS in LUAD patients at 1, 2, and 3 years.


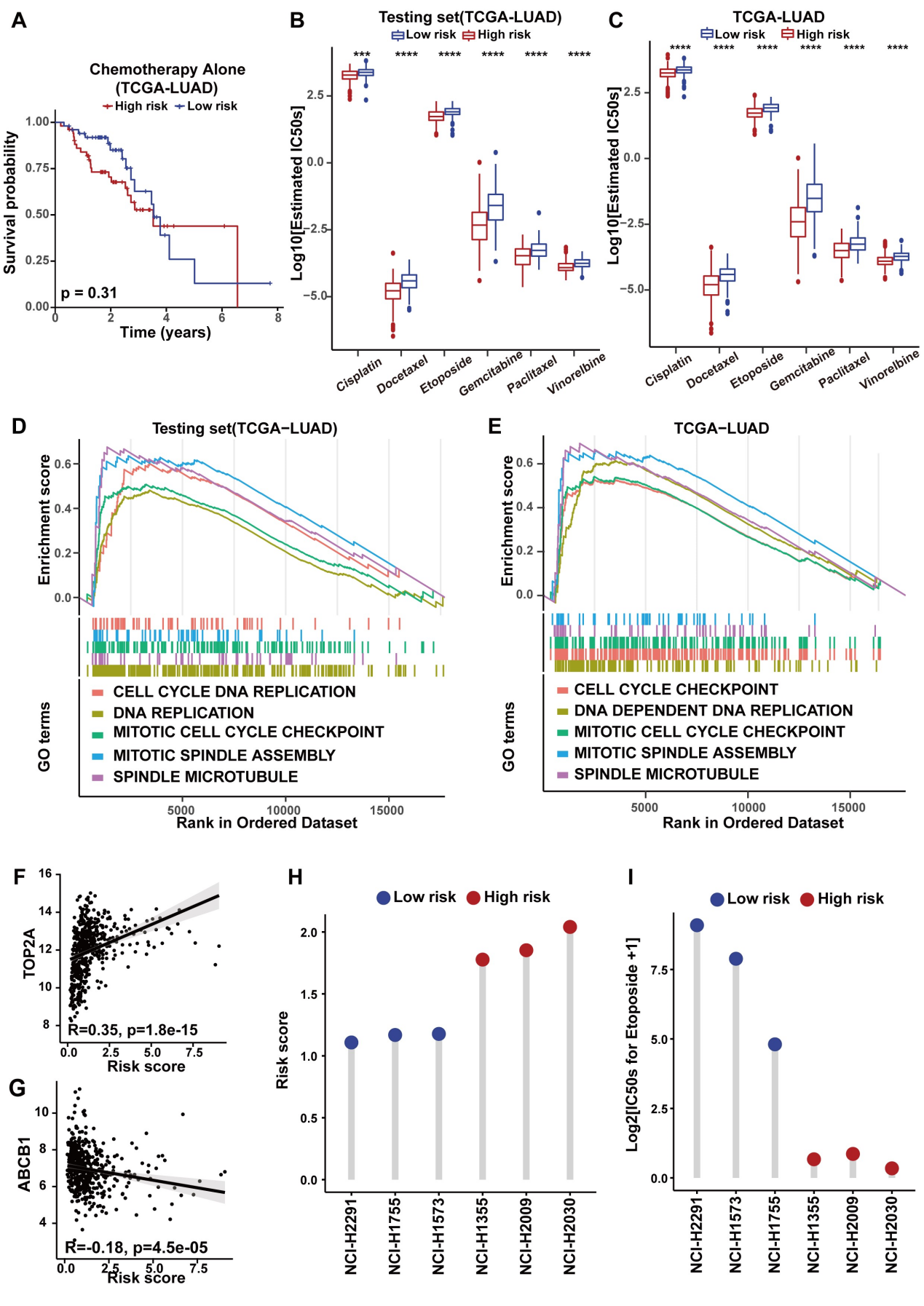


**Additional file 4: Figure S3. Prediction of chemotherapy response level in LUAD patients.**

(**A**) Kaplan-Meier survival curve for LUAD patients receiving chemotherapy alone in TCGA. (**B-C**) Boxplots reflect the differences in the degree of response to chemotherapy drugs between patients in the high-risk and low-risk groups in the TCGA-LUAD testing set (B) and TCGA-LUAD set (C). ***P < 0.001 and ****P < 0.0001. (**D-E**) GSEA analysis shows signaling pathways significantly enriched in patients in the high-risk groups in the TCGA-LUAD testing set (D) and TCGA-LUAD set (E). (**F**) Pearson correlation coefficient between risk score and TOP2A expression. (**G**) Pearson correlation coefficient between risk score and ABCB1 expression. (**H**) Risk scores of LUAD cell lines in the high-risk and low-risk groups. (**I**) Drug sensitivity to Etoposide of LUAD cell lines in the high-risk and low-risk groups.


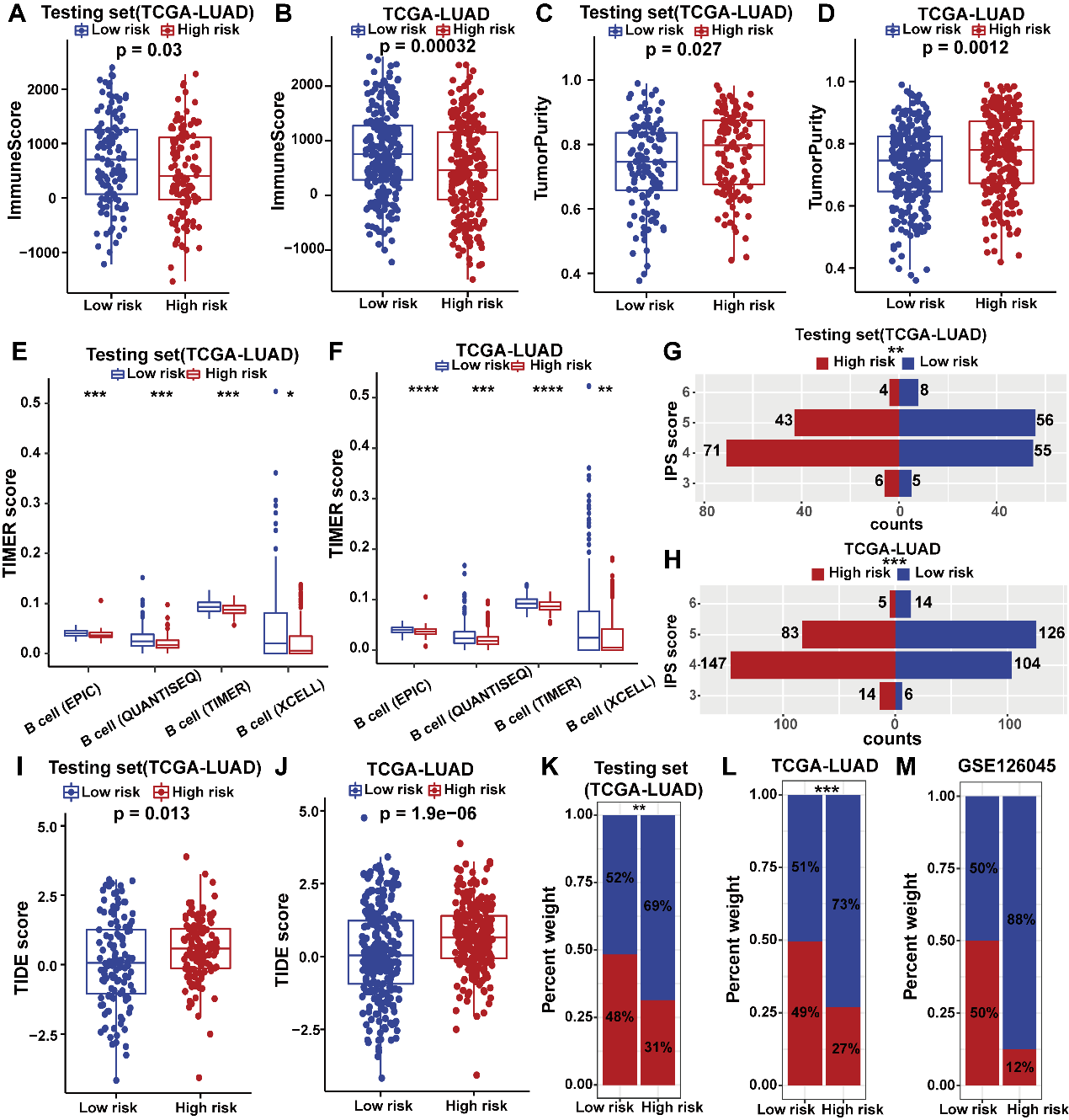
**Additional file 4: Figure S4.** **The GSAGI predicts the immune characteristics of LUAD.**

(**A-B**) ESTIMATE algorithm evaluates the immune score of patients in the high-risk and low-risk groups in the TCGA-LUAD testing set (A) TCGA-LUAD set (B). (**C-D**) ESTIMATE algorithm evaluates the tumor purity of patients in the high-risk and low-risk groups in the TCGA-LUAD testing set (C) TCGA-LUAD set (D). (**E-F**) Four algorithms in the TIMER database assess the differences in the degree of infiltration of B cells between patients in the high-risk and low-risk groups in the TCGA-LUAD testing set (E) TCGA-LUAD set (F). *P< 0.05, **P < 0.01, ***P < 0.001 and ****P < 0.0001. (**G-H**) Two-way bar graphs show IPS for patients in the high-risk and low-risk groups in the TCGA-LUAD testing set (G) and TCGA-LUAD set (H). **P < 0.01 and ***P < 0.001. (**I-J**) Comparison of TIDE scores between patients in the high-risk and low-risk groups in the TCGA-LUAD testing set (I) TCGA-LUAD set (J). (**K-L**) The percentage bar graph compares the different response statuses of patients receiving immunotherapy in the high-risk and low-risk groups in the TCGA-LUAD testing set (K) TCGA-LUAD set (L). **P < 0.01 and ***P < 0.001. Red indicates that the patient responded to ICI treatment, and blue indicates non-response. (**M**) The percentage bar chart compares the response status of patients in the high-risk and low-risk groups receiving immunotherapy in the GSE126045 dataset. Red indicates that the patient responded to ICI treatment, and blue indicates non-response.


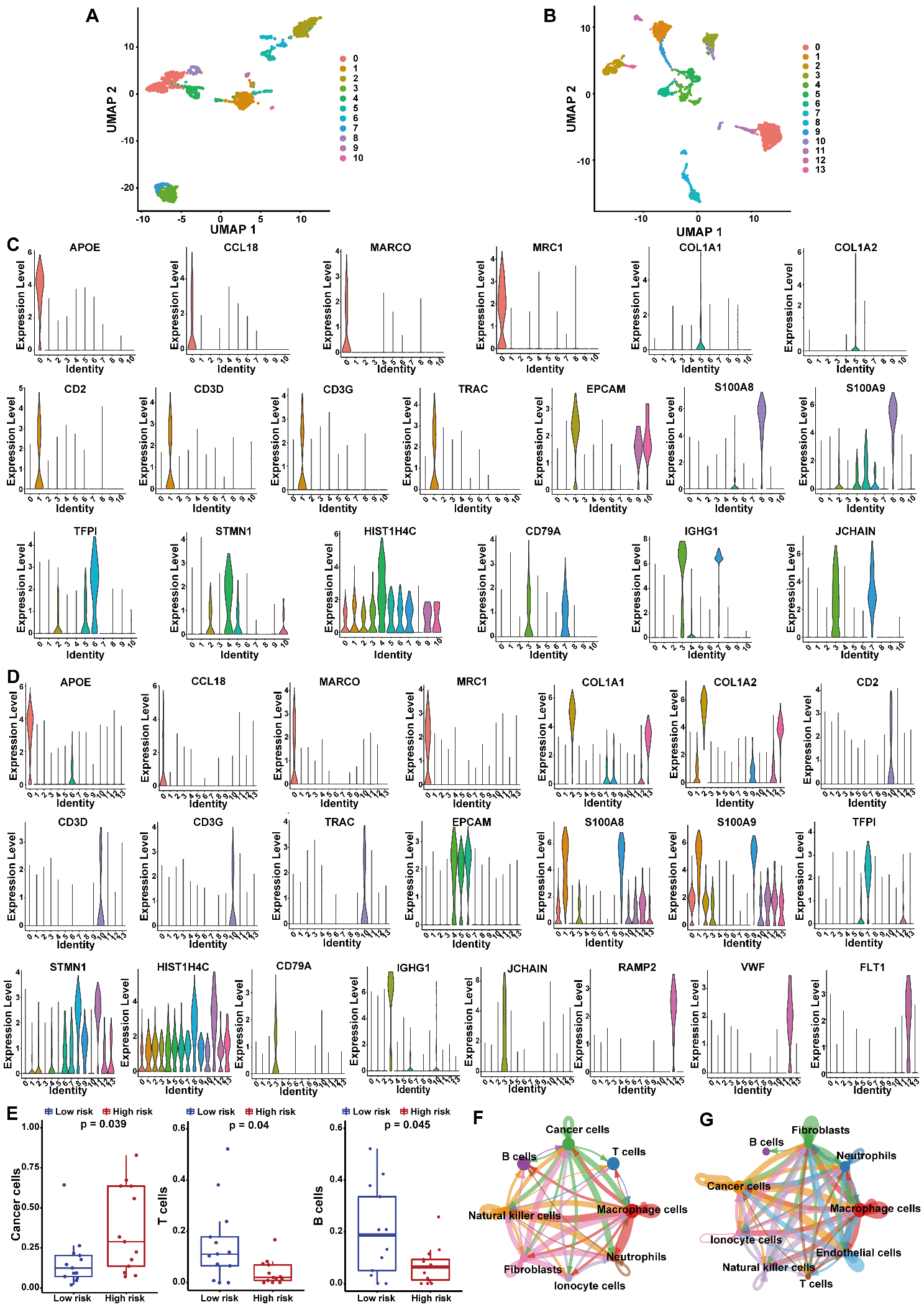


**Additional file 4: Figure S5.**  **Cellular clustering and expression of marker genes at the single-cell level.**

(**A-B**) UMAP shows cellular clustering in low (A) and high (B) subgroups. (**C**) Expression of marker genes in individual cell clusters in patients in the low subgroup. (**D**) Expression of marker genes for individual cell clusters in patients in the high subgroup. (**E**) Differences in the expression of tumor cells, T-cells, and B-cells in low and high subgroups. (**F-G**) Interaction of all cells in TME of low (F) and high (G) subgroups.

**Additional file 4: Figure S6.** **Impact of characterized genes in GSAGI on survival in LUAD patients.**
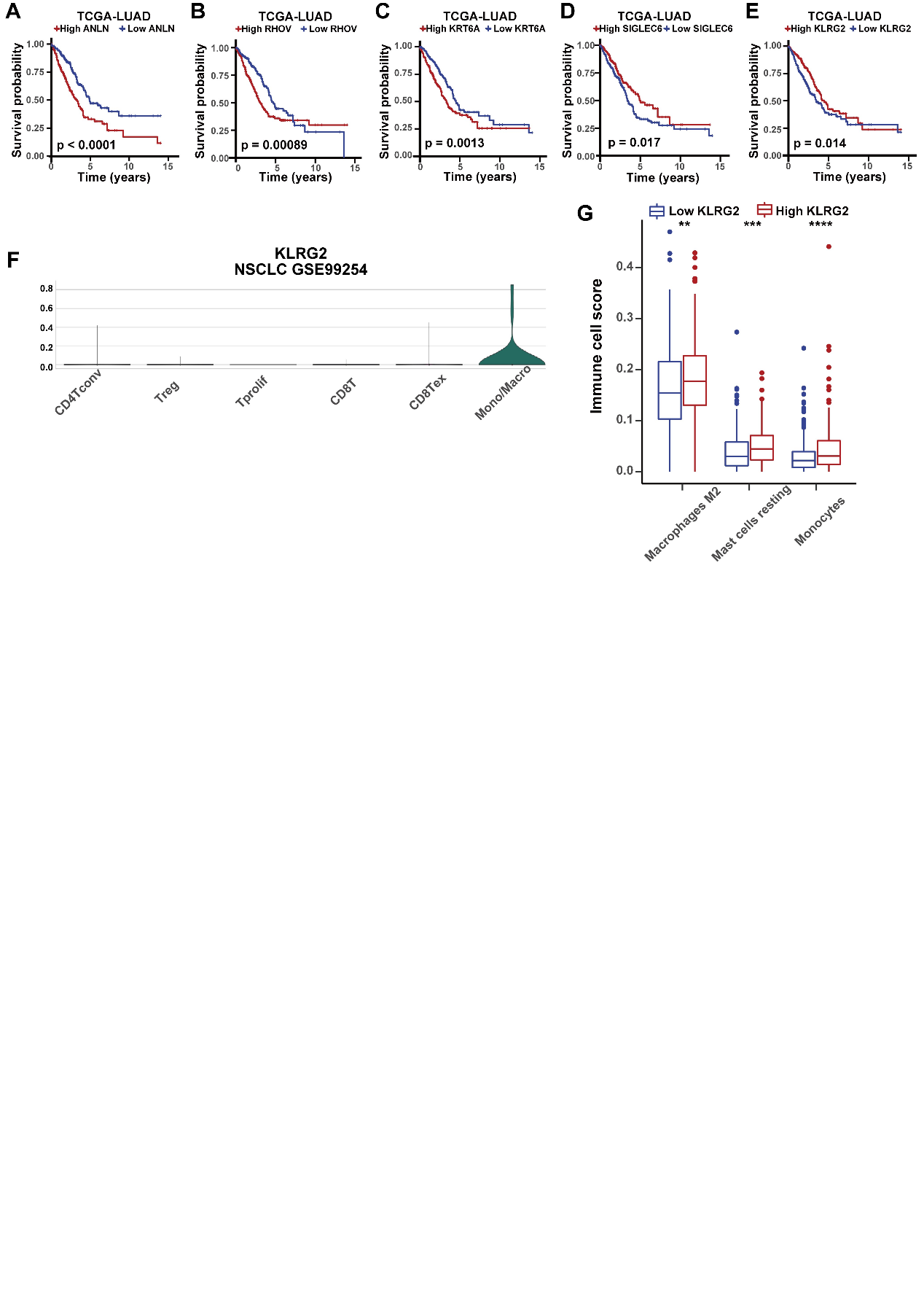


(**A-E**) Kaplan-Meier survival curves were plotted in TCGA-LUAD with the median expression of ANLN, RHOV, KRT6A, SIGLEC6, and KLRG2 to distinguish patients in the high and low expression groups. (**F**) Expression of KLRG2 in different cell types in the NSCLC dataset GSE99254 from the TISCH2 database. (**G**) CIBERSORT algorithm to assess the infiltration of different types of immune cell infiltration in patients in the high and low KLRG2 express.
